# Potassium Channel KCNH1 Activating Variants Cause Altered Functional and Morphological Ciliogenesis

**DOI:** 10.1007/s12035-022-02886-4

**Published:** 2022-05-31

**Authors:** Giulia Napoli, Noemi Panzironi, Alice Traversa, Caterina Catalanotto, Valentina Pace, Francesco Petrizzelli, Agnese Giovannetti, Sara Lazzari, Carlo Cogoni, Marco Tartaglia, Massimo Carella, Tommaso Mazza, Antonio Pizzuti, Chiara Parisi, Viviana Caputo

**Affiliations:** 1 Institute of Biochemistry and Cell Biology, CNR-National Research Council, Monterotondo Scalo, Rome, Italy; 2 Laboratory of Clinical Genomics, Fondazione IRCCS Casa Sollievo Della Sofferenza, San Giovanni Rotondo (FG), Italy; 3 Department of Molecular Medicine, Sapienza University of Rome, Rome, Italy; 4 Department of Experimental Medicine, Sapienza University of Rome, Rome, Italy; 5 Unit of Bioinformatics, Fondazione IRCCS Casa Sollievo Della Sofferenza, San Giovanni Rotondo (FG), Italy; 6 Genetics and Rare Diseases Research Division, Ospedale Pediatrico Bambino Gesù, IRCCS, Rome, Italy; 7 Research Unit of Medical Genetics, Fondazione IRCCS Casa Sollievo Della Sofferenza, San Giovanni Rotondo (FG), Italy

**Keywords:** KCNH1, Potassium channel, Primary cilium, Neurodevelopmental disorder, Epilepsy, SHH pathway

## Abstract

The primary cilium is a non-motile sensory organelle that extends from the surface of most vertebrate cells and transduces signals regulating proliferation, differentiation, and migration. Primary cilia dysfunctions have been observed in cancer and in a group of heterogeneous disorders called ciliopathies, characterized by renal and liver cysts, skeleton and limb abnormalities, retinal degeneration, intellectual disability, ataxia, and heart disease and, recently, in autism spectrum disorder, schizophrenia, and epilepsy. The potassium voltage-gated channel subfamily H member 1 (*KCNH1*) gene encodes a member of the EAG (ether-à-go-go) family, which controls potassium flux regulating resting membrane potential in both excitable and non-excitable cells and is involved in intracellular signaling, cell proliferation, and tumorigenesis. *KCNH1* missense variants have been associated with syndromic neurodevelopmental disorders, including Zimmermann-Laband syndrome 1 (ZLS1, MIM #135500), Temple-Baraitser syndrome (TMBTS, MIM #611816), and, recently, with milder phenotypes as epilepsy. In this work, we provide evidence that KCNH1 localizes at the base of the cilium in pre-ciliary vesicles and ciliary pocket of human dermal fibroblasts and retinal pigment epithelial (hTERT RPE1) cells and that the pathogenic missense variants (L352V and R330Q; NP_002229.1) perturb cilia morphology, assembly/disassembly, and Sonic Hedgehog signaling, disclosing a multifaceted role of the protein. The study of KCNH1 localization, its functions related to primary cilia, and the alterations introduced by mutations in ciliogenesis, cell cycle coordination, cilium morphology, and cilia signaling pathways could help elucidate the molecular mechanisms underlying neurological phenotypes and neurodevelopmental disorders not considered as classical ciliopathies but for which a significant role of primary cilia is emerging.

**Supplementary Information:**

The online version contains supplementary material available at 10.1007/s12035-022-02886-4.

## Introduction

The primary cilium is a non-motile sensory organelle that extends from the surface of most vertebrate cells and transduces signals from extracellular stimuli to cell pathways regulating proliferation, differentiation, migration, and tissue morphology [1]. The primary cilium is composed of an axoneme structure enclosed by a bilayer lipid membrane, protruding from the apical surface of a modified centriole, the basal body, and comprising a radial array of nine microtubule pairs lacking the central doublet (9 + 0 structure) [2]. At the base of the primary cilium, protein trafficking is regulated through the transition fiber, anchoring the axoneme to the ciliary membrane [3–5].

The vesicles’ trafficking machinery is pivotal for both ciliogenesis and proteins’ movement within the cilium. In most cell types, including neurons and hTERT-immortalized retinal pigment epithelial cell line (hTERT RPE-1), the primary site of vesicles’ formation for both exo- and endocytosis localizes at the basal part of the primary cilium, within an invagination of the plasma membrane known as the ciliary pocket [6].

The dual function of centriole in the basal body and the centrosome allows to couple ciliogenesis and ciliary signals to cell cycle, leading to cilia assembly in the post-mitotic G0/G1 phases of the cell cycle and cilia disassembly before mitosis [7].

Mutations in genes impairing ciliary biogenesis and functions cause a group of heterogeneous clinical disorders called ciliopathies, characterized by a phenotype including renal and liver cysts, skeleton and limb abnormalities, retinal degeneration, intellectual disability (ID), ataxia, and heart disease, reflecting the complexity of ciliogenesis and the crucial role of primary cilia in cell development and physiology [8]. The role of primary cilia function has also been suggested in neurodevelopmental processes through the interplay of several genes whose alterations have been proposed to underlie autism spectrum disorder (ASD), schizophrenia, ID [9–12], and epilepsy [13, 14], which had not been previously implicated in classical ciliopathies.

The potassium voltage-gated channel subfamily H member 1 (Kv10.1, H-Eag, *KCNH1*, MIM *603305) gene encodes a member of the EAG (ether-à-go-go) family. This member is a pore-forming subunit of a voltage-gated non-inactivating delayed rectifier potassium channel that is composed of the assembly of four subunits.

*KCNH1* is mainly expressed in the adult central nervous system (GTEx, www.gtexportal.org) and plays a role in controlling K^+^ flux that regulates resting membrane potential in both excitable and non-excitable cells [15, 16] and is activated at the onset of myoblast differentiation [17]. The channel is also involved in cell proliferation and differentiation processes, particularly in adipogenic and osteogenic differentiation in bone marrow-derived mesenchymal stem cells (MSCs) [18].

The protein has been localized at the plasma membrane [19–21], the inner nuclear membrane [22], and intracellular vesicles [23]. It has been recently detected in the primary cilium area in hTERT RPE-1 cells and in mouse embryonic fibroblasts [24], where it participates in ciliogenesis through the interaction with proteins involved in ciliary regulation, as Rabaptin-5, cortactin, and Hypoxia-inducible factors [24].

*KCNH1* ectopic expression has been reported in most human cancers [25, 26], and its function has also been associated with intracellular signaling, cell proliferation, and tumorigenesis in a way that appears unrelated to its role in ion permeation [26]. Recent studies reported causative de novo heterozygous missense mutations involving this channel in developmental diseases that include Zimmermann-Laband syndrome 1 (ZLS1, MIM #135500) [27–29], Temple-Baraitser syndrome (TMBTS, MIM #611816) [30–32], syndromic intellectual disability [33] and syndromic developmental delay, hypotonia and seizures [34]. All those phenotypes are characterized by neurological manifestations, including ID and epilepsy, suggesting an important role for *KCNH1* in human neurodevelopment. More recent studies expanded the phenotype spectrum of *KCNH1*-related encephalopathies to subjects with severe intellectual disability, mild extra-neurological phenotype, and lacking the distinctive features of TMBTS and ZLS1 [35–39].

The biological mechanisms altered by the pathogenic *KCNH1* mutations, which lead to developmental phenotypes, are still poorly understood. Most functional studies relied on electrophysiological approaches to characterize the functional effect of pathogenic missense mutations and disclosed a gain of function mechanism causing altered properties of the channel [27, 30]. However, little is known about channel dysfunctions related to its subcellular localization, trafficking, and interactions.

In this work, we studied KCNH1 protein subcellular localization, focusing on the primary cilium structure in different cell types, by confocal microscopy. As the mechanisms of KCNH1 related to ciliary functions remain largely unknown, and the functional effects of pathogenic variants have been only partially investigated, we evaluated cilium assembly dynamics, cilium morphology, and Sonic Hedgehog (SHH) pathway activation in wild-type cells and with selected pathogenic variants.

We provided evidence that KCNH1 localizes at the base of the cilium in pre-ciliary vesicles and ciliary pocket compartments that regulate ciliogenesis dynamics. Moreover, we investigated cilia assembly/disassembly, morphology, and SHH signaling, further supporting a functional role of K^+^ channels in ciliary processes and shedding light into the molecular bases of K^+^ channelopathies.

## Material and Methods

### Cell Culture and Treatments

Human dermal fibroblasts (HDF) cells were obtained from ATCC (PCS-201–012, Sigma-Aldrich). Primary skin fibroblasts were obtained from subcutaneous biopsies of patients carrying pathogenic *KCNH1* variants c.1054C > G, p.L352V [27] and c.989G > A, p.R330Q [28]. The selected pathogenic variants were chosen as they represent two available patient-derived samples, and they are reported according to *KCNH1* transcript variant 2 (RefSeq: NM_002238.4; NP_002229.1). We re-analyzed the whole exome of both patients to identify further putative deleterious rare variants altering the coding sequence or the splicing mechanism. We did not identify any additional putative candidate variants that could be associated with phenotypes.

Fibroblasts and hTERT RPE-1 cell lines were cultured in Dulbecco’s modified Eagle’s medium (DMEM) and DMEM/F-12, respectively, both supplemented with 10% heat-inactivated fetal bovine serum (FBS, EuroClone) and 1% penicillin–streptomycin, at 37 °C with 5% CO_2_.

For KCNH1 localization, cilia count, and morphology analyses, fibroblasts and hTERT RPE-1 cells were plated onto coverslips and cultured in complete medium for 24 h. Then, cells were starved in a serum-free medium for 10 min, 20 min, and 48 h before immunofluorescence (IF) analysis. To induce cilium disassembly, the serum was reintroduced for 1, 2, and 4 h after 48 h of starvation, and cells were fixed with 4% paraformaldehyde (PFA).

To induce Sonic Hedgehog (SHH) signaling pathway activity, wild-type and mutant fibroblasts were starved in serum-free media for 24 h and then treated with a Smoothened, Frizzled Class Receptor (SMO) agonist (SAG; Sigma-Aldrich), 100 nM for 24 h.

### Immunofluorescence Analysis

Fibroblasts and hTERT RPE-1 cells were washed with phosphate buffered saline (PBS) and fixed using 4% PFA followed by permeabilization with PBS + 0.1% TRITON X-100, blocked in blocking buffer (3% bovine serum albumin—BSA—in PBS), and subjected to incubation with primary and secondary antibodies (Supplementary Table 1). Nuclei were stained with DAPI (Sigma-Aldrich), and coverslips were mounted using Fluoromount (Sigma-Aldrich).

### Image Acquisition and Analysis

Confocal images were sequentially acquired by Olympus’ PLAPON 60X OSC2 super-corrected objective confocal apparatus. Sequential 0.5-μm-thick z-stacked sections were imaged through the entire cell profile using a 60 × objective lens and were used to create maximum intensity projections (MIPs) and processed with Fiji (National Institute of Health). All images were acquired with the same laser intensity before the analysis in Fiji.

For primary cilia count, the proportion of ciliated cells in a single field was determined by counting the number of cilia and the number of nuclei, and then it was expressed as the percentage of the total cell population. This analysis was performed for 6 different fields in three different experiments. Statistical significance was determined by unpaired Student’s *t* test (*p* < 0.05).

Cilia lengths were measured using straight, segmented, or freehand lines tool of fluorescent axoneme marker in maximum Z intensity projected images in Fiji. Cilia were categorized based on mean + / − standard deviation (sd) values calculated of cilia of control (wild-type cells), as follows: short cilia < 2.47 μm (mean-sd); normal cilia 2.47–4.05 μm; long cilia > 4.05 μm (mean + sd) [40].

Colocalization was quantified using the Pearson’s correlation coefficient (PCC) within the selected areas of the images. Briefly, PCC is used to quantify colocalization, with values ranging from 1, for two images whose fluorescence intensities are perfectly and linearly positively related, to − 1, for two images whose fluorescence intensities are perfectly but inversely related. Values close to zero reflect distributions of probes that are uncorrelated. Imaging data from the experiments were analyzed using the Coloc 2 plugin of the Fiji distribution of ImageJ software (http://imagej.net/Coloc_2). Before applying a mask and running the plugin, individual areas were selected as regions of interest (ROIs). After running the plugin, PCC reported in the ImageJ Log window was recorded for each cell and reported as mean + standard error mean (sem) on 30 cells.

### Quantitative RT-PCR (qPCR)

Total RNA from dermal fibroblasts of wild-type and carrying *KCNH1* pathogenic variants was extracted using TRIZOL reagent (Invitrogen, Life Technologies) according to the manufacturer’s instructions. RNA quantity was determined with the Nanodrop 1000 System (ThermoFisher Scientific). qPCR was performed in triplicate using SYBR Green (Applied Biosystems) after reverse transcription of 1 µg of total RNA using the High-Capacity cDNA Reverse Transcription Kit (Applied Biosystems) with random primers, following the manufacturer’s instructions. Relative gene expression was calculated by the ΔΔC_T_ method relative to glyceraldehyde-3-phosphate dehydrogenase (*GAPDH)* expression levels. Primers for quantitative analysis are listed in Supplementary Table 2.

## Results

### KCNH1 Localizes to Membrane Subdomains at the Base of the Primary Cilia of hTERT RPE-1 Cells

We examined KCNH1 localization through immunofluorescence/confocal microscopy analysis in 48 h serum-starved hTERT RPE-1 cells to induce primary cilia assembly.

**Fig. 1:**
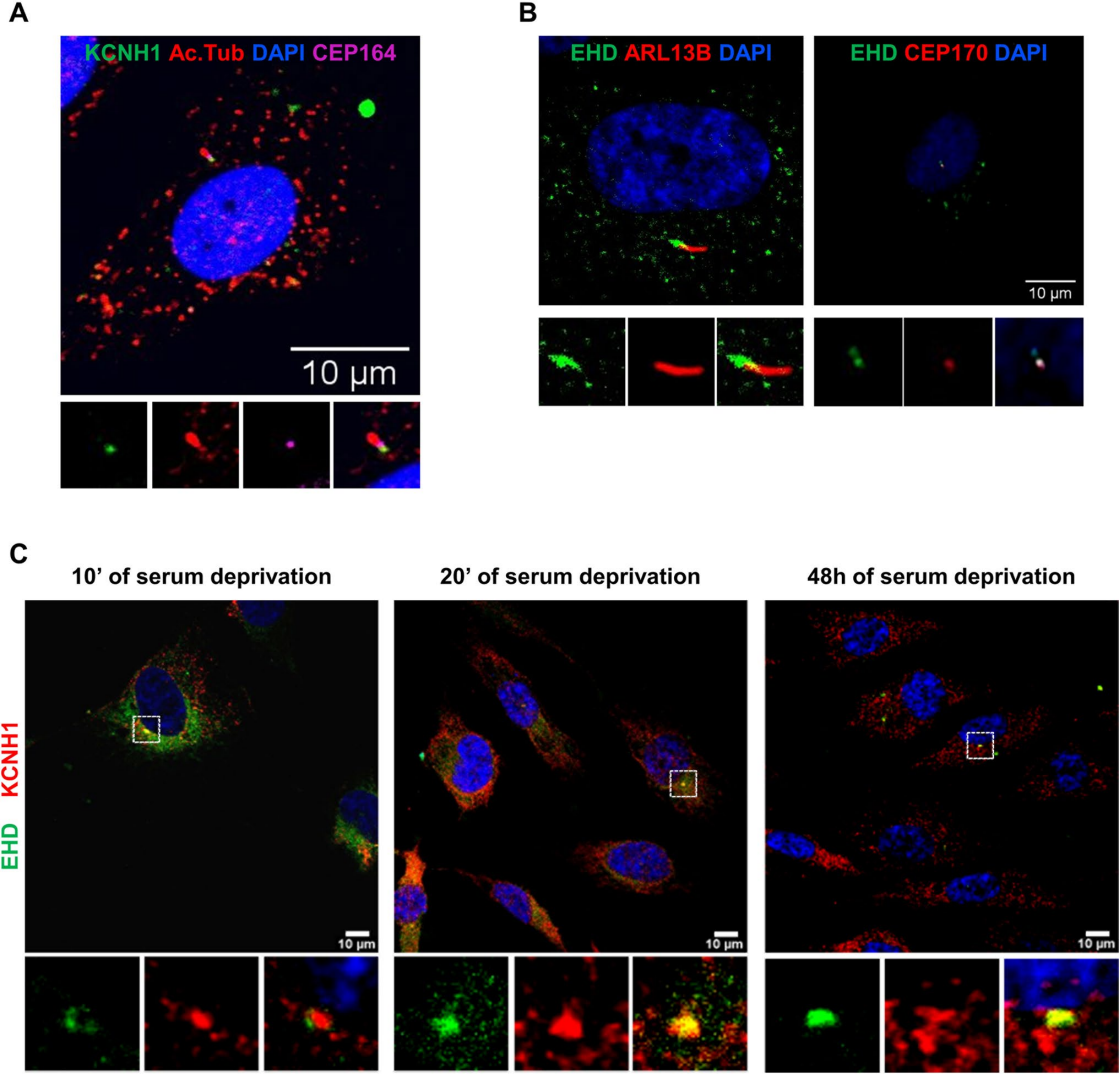
KCNH1 localizes to the primary cilia pocket of hTERT RPE-1 cells. (**a**) Representative images of immunofluorescence (IF) staining detecting KCNH1 localization (green) to the base of the primary cilium. hTERT RPE-1 cells were serum-starved for 48 h and analyzed by IF microscopy. DAPI (blue) was used to visualize nuclei, acetylated tubulin (Ac. Tub) staining (red) detected primary cilium axoneme and CEP164 (pink) the centrosome. Images are maximum intensity projections of z-stacks. Scale bars 10 µm. (**b**) Representative images of EHD family members 1–4 (green) localization to the ciliary pocket. ARL13B (red) and CEP170 (red) detected respectively primary cilium axoneme and the centrosome. Scale bars 10 µm. (**c**) Representative images of IF microscopy analysis of colocalization (yellow) between KCNH1 (red) and EHD (green) after 10 min, 20 min, and 48 h of serum deprivation. Scale bars 10 µm

The cilium structure was defined by the staining of acetylated alpha-tubulin (Ac. Tub), an axoneme marker. KCNH1 was detected at the base of the primary cilium, close to the cilium base marker, Centrosomal Protein 164 (CEP164), a centrosomal protein required for primary cilia assembly (Fig. 1a), confirming previous results localizing KCNH1 in the centrosomal component [24]. Immunofluorescence staining using antibody for C-terminal Eps15 Homology Domain (EHD) family members 1–4, markers of pre-ciliary membranes and the ciliary pocket (Fig. 1b), specifically localizes KCNH1 to membrane subdomains at the base of primary cilia of hTERT RPE-1 cells (Fig. 1c), in addition to a diffuse peripheral staining, consistent with the previously described pattern indicating its localization at the plasma membrane and endocytic vesicles [41]. These results define the localization of KCNH1 within the primary cilium, for the first time to our knowledge, in the ciliary pocket structure and pre-ciliary vesicles since the early phases of primary cilium assembly (Fig. 1c).

### Effect of Pathogenic KCNH1 Variants on Primary Cilia Localization

We evaluated whether disease-causing mutations could affect the ciliary protein localization. To this aim, we analyzed fibroblasts of patients carrying KCNH1^L352V^ [27] and KCNH1^R330Q^ proteins (NP_002229.1) [28], after 48 h of serum starvation. As expected, we detected KCNH1 marked distribution in proximity of Golgi and early endosomal compartment (Supplementary Fig. 1) [41].

**Fig. 2:**
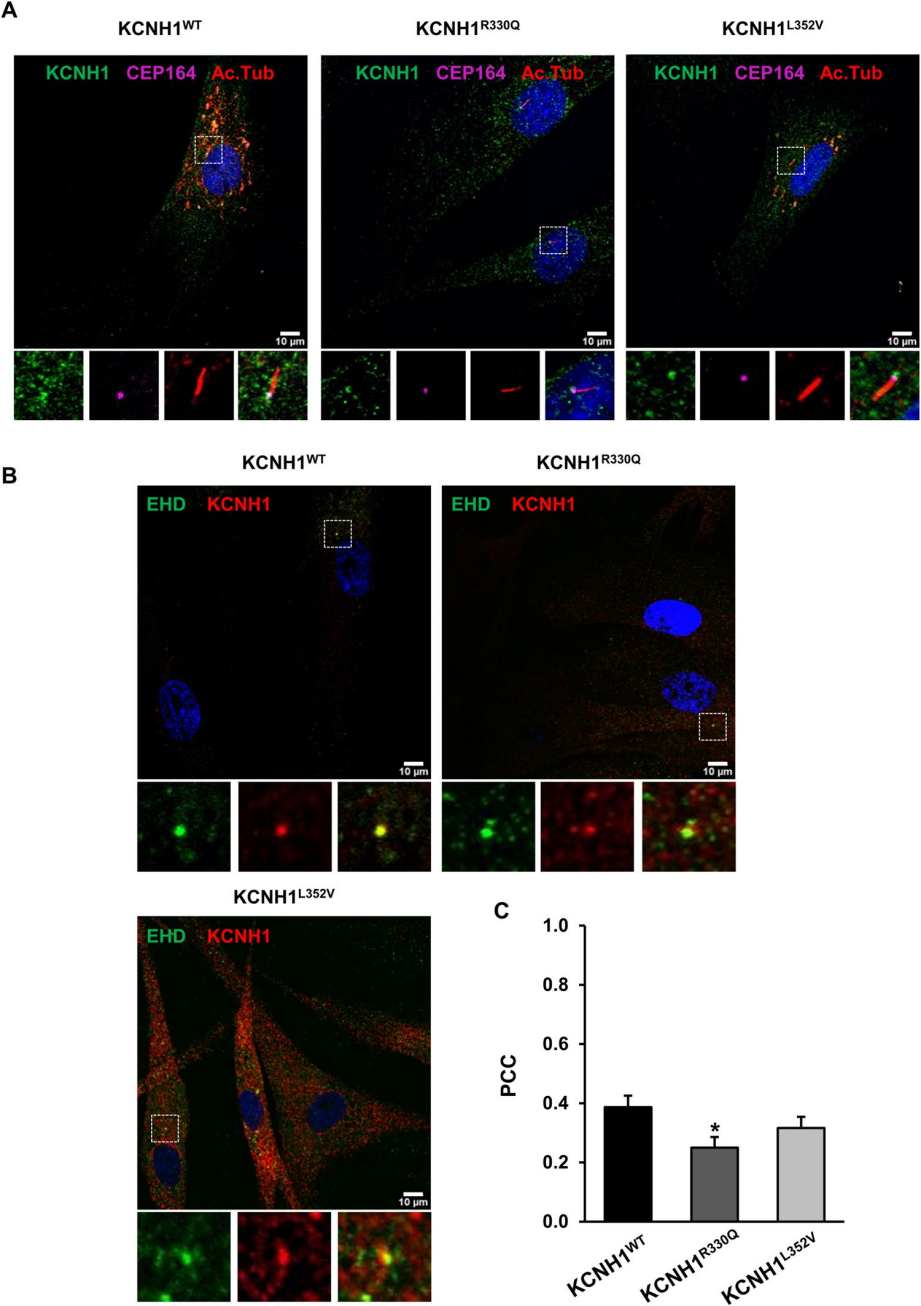
Disease-causing KCNH1 missense mutations affect ciliary pocket localization. Wild-type (KCNH1^WT^) and patients’ fibroblasts carrying KCNH1^R330Q^ and KCNH1^L352V^ mutations were serum-starved for 48 h and analyzed by IF. DAPI (blue) was used to visualize nuclei, acetylated tubulin (Ac. Tub) staining detected ciliary axoneme, CEP164 the centrosome and EHD the ciliary pocket. Images are maximum intensity projections of z-stacks. Scale bars 10 µm. (**a**) Representative images of IF staining detecting KCNH1^WT^, KCNH1^R330Q^, and KCNH1^L352V^ (green) localization to the base of the primary cilium (CEP164 pink, Ac. Tub. red). (**b**) Representative images of colocalization (yellow) between KCNH1 (red) and EHD (green). (**c**) Quantification of b. For each experimental condition, Pearson’s correlation coefficient (PCC) was calculated within the areas of colocalization using Fiji. PCC was recorded for each cell and reported as mean + sem on 30 cells. Differences between two groups were analyzed by unpaired Student’s *t* test. *p = 0.01541 vs wild-type

Additionally, both wild-type and mutant KCNH1 proteins accumulate at the primary cilium base of fibroblasts, just below the centrosome protein CEP164 (Fig. 2a, Supplementary video), and in correspondence to the ciliary pocket area and pre-ciliary vesicles, colocalizing with EHD proteins (Fig. 2b). Localization of both mutants at the primary cilium base was also confirmed for Flag-KCNH1 constructs ectopically overexpressed in hTERT RPE-1 cells (Supplementary Fig. 2).

Pearson’s correlation coefficients (PCCs) indicate an association between KCNH1 and EHD (PCC = 0.39), in control cells, while for mutants, a mild decrease of KCNH1^L352V^ (PCC = 0.32) and a more pronounced and significant decrease of KCNH1^R330Q^ (PCC = 0.25, *p* = 0.01541) to ciliary pocket (Fig. 2c) were observed. These results suggest that pathogenic *KCNH1* variants could moderately affect protein localization to the primary cilium.

### KCNH1^R330Q^ and KCNH1^L352V^ Mutants Cause Abnormal Ciliogenesis and Cell Cycle Defects

**Fig. 3:**
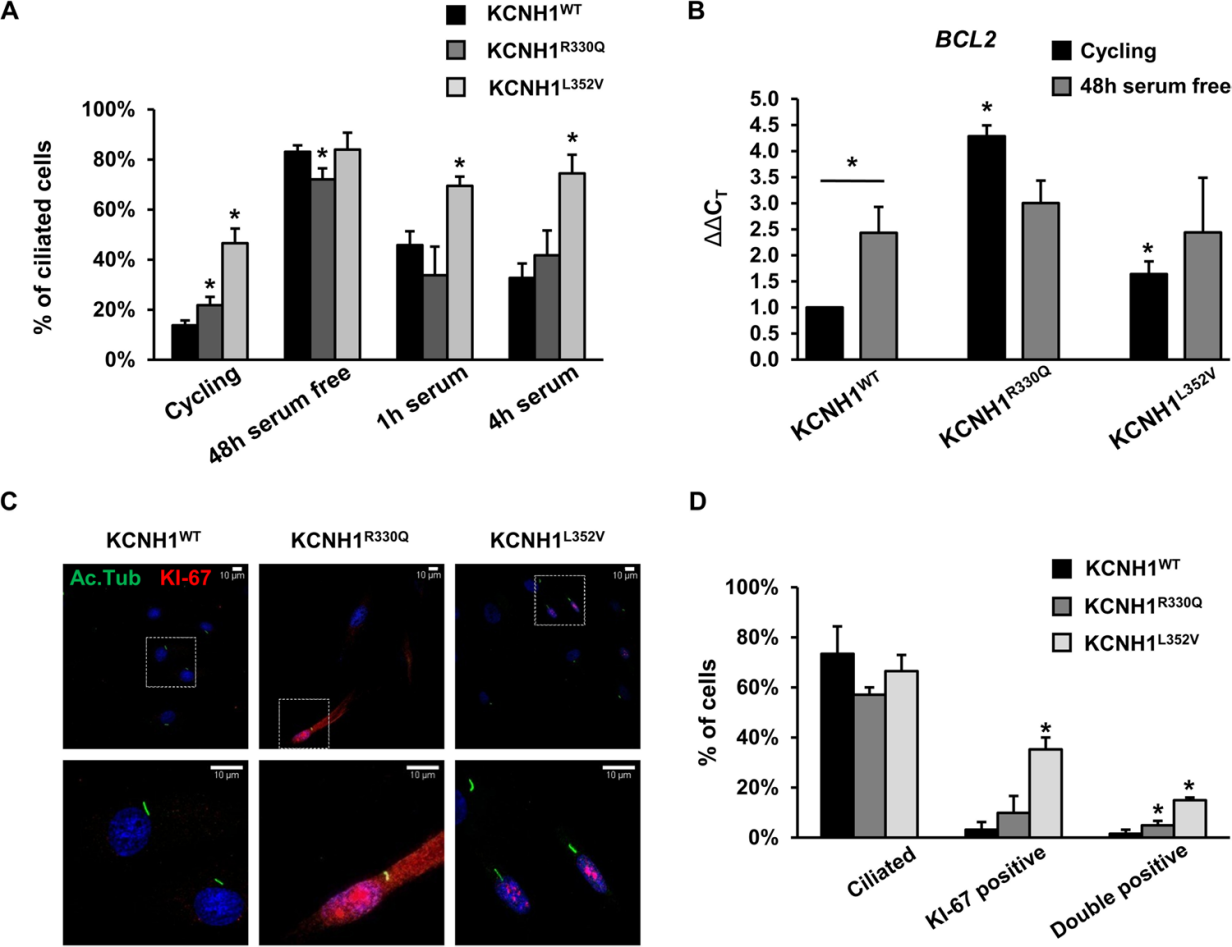
KCNH1 mutations cause abnormal ciliogenesis and cell cycle defects. (**a**) Quantification of the number of ciliated cells in wild-type and patients’ fibroblasts carrying KCNH1^R330Q^ and KCNH1^L352V^ mutations. (**b**) *BCL2* mRNA expression was measured by qPCR from wild-type and patients’ fibroblasts carrying KCNH1^R330Q^ and KCNH1^L352V^ mutations. *GAPDH* was used as a reference gene for normalization. (**c**) Representative images of IF analysis of Ki-67 (red) and acetylated tubulin (Ac. Tub) (green) of 48 h serum-starved wild-type and patients’ fibroblasts carrying KCNH1^R330Q^ and KCNH1^L352V^ mutations. Scale bars 10 µm. (**d**) Quantification of c. Data are represented as mean + sem. Differences between two groups were analyzed by unpaired Student’s *t* test. *p < 0.05 is reported vs the same condition of wild-type unless otherwise indicated with bars

We investigated the role of KCNH1 in ciliogenesis and the effect introduced by the R330Q and L352V amino acid substitutions. As expected, in fibroblasts expressing the wild-type protein, we observed an increase of cilia number in serum-free condition and a decrease after serum stimulation (1 h and 4 h) (Fig. 3a). Similar dynamics were observed for both patients’ fibroblasts (Fig. 3a). When we compared cilia numbers among wild-type and mutant cells, we observed that in cycling conditions, both mutants showed a significantly higher number of ciliated cells (Fig. 3a). Upon serum removal, comparable numbers were observed between wild-type and KCNH1^L352V^ mutant fibroblasts, while KCNH1^R330Q^ mutants showed a small but significant reduction of ciliated cells. After 1 h and 4 h of serum stimulation, a higher number of cilia was observed in KCNH1^L352V^ fibroblasts, further suggesting a slower dynamics of primary cilium disassembly (Fig. 3a).

As ciliogenesis is synchronized to cell cycle [42], we evaluated the effect of *KCNH1* missense mutations on the expression of BCL2 apoptosis regulator (*BCL2*), a cell cycle regulator with anti-proliferative functions which facilitates the arrest in G0 [43]. Gene expression analysis showed an increase of *BCL2* transcript in KCNH1^R330Q^ and KCNH1^L352V^ cycling fibroblasts compared to wild-type cells (Fig. 3b; *p* < 0.05 in both comparisons). The *BCL2* expression profile of mutant fibroblasts showed a trend similar to that of quiescent serum-starved control fibroblasts (Fig. 3b). These results suggest that *KCNH1* mutations may cause defects in cell cycle progression.

To test the hypothesis of a loss of coordination between ciliogenesis and cell cycle, we evaluated the number of ciliated-Ki-67 (marker of proliferation Ki-67)-positive cells, a marker of cycling cells [44] (Fig. 3c). Both KCNH1^R330Q^ and KCNH1^L352V^ mutants increased the fraction of cycling (Ki-67-positive) cells, and they also significantly increased the percentage of ciliated-Ki-67-positive cells (Fig. 3d). In detail, R330Q substitution increased by approximately 3 times (*p* < 0.05) and L352V about 9 times ciliated-Ki-67-positive cells (*p* < 0.05), respectively. These results suggest that *KCNH1* mutations could reduce the coupling between ciliogenesis and cell cycle.

### KCNH1^R330Q^ and KCNH1^L352V^ Mutations Alter Cilia Length and Morphology and Affect Ciliary Transport

**Fig. 4:**
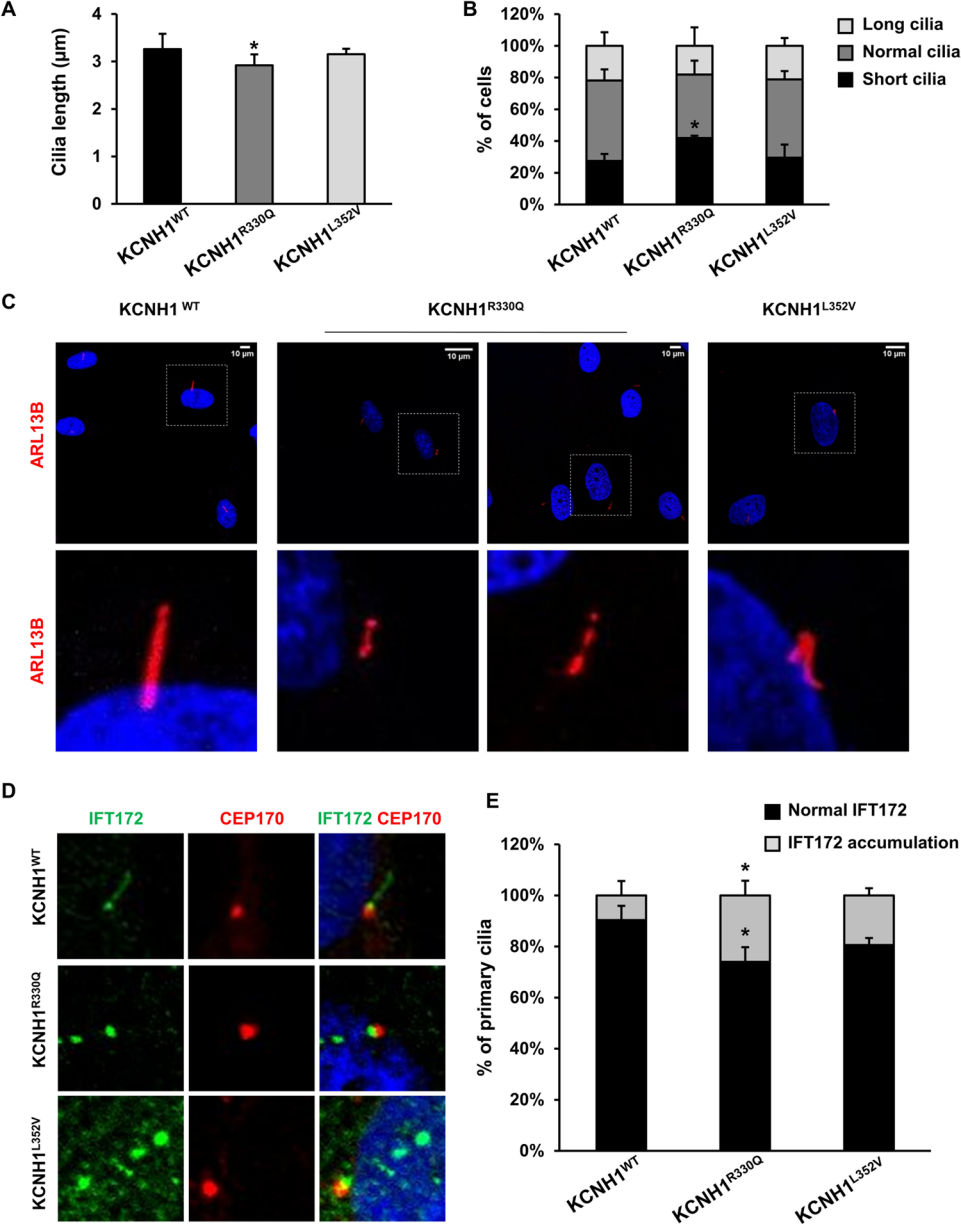
KCNH1 mutations alter cilia length and morphology and affect ciliary transport. (**a**) Graph of cilia length measurements of KCNH1^WT^, KCNH1^R330Q^, and KCNH1^L352V^ fibroblasts. (**b**) Percentage of cells with cilia of different lengths. *p = 0.03511 vs wild-type. (**c**) Representative images of morphological anomalies of primary cilia of KCNH1^R330Q^ and KCNH1^L352V^ fibroblasts stained with ARL13B antibody (red). Scale bars 10 µm. (**d**) Representative image of IFT172 staining (green) of KCNH1^WT^, KCNH1^R330Q^, and KCNH1^L352V^ fibroblasts. CEP170 (red) was used to visualize the primary cilium base. Scale bars 10 µm. (**e**) Quantification of d. Data are represented as mean + sem. Differences between two groups were analyzed by unpaired Student’s *t* test. *p < 0.05 is reported vs the same condition of wild-type

We tested cilia length and morphology of wild-type and mutant fibroblasts, and we found significant differences in KCNH1^R330Q^ mutant cells. Cilia of wild-type cells were 3.26 ± 0.79 μm in length, whereas mutant cells had cilia of 2.91 ± 0.57 μm and 3.15 ± 0.23 μm, in KCNH1^R330Q^ and KCNH1^L352V^ mutant fibroblasts, respectively (Fig. 4a). Specifically, cilia of KCNH1^R330Q^ mutant cells were 10.7% shorter than controls (*p* < 0.05), and the number of cells with short cilia (i.e., < 2.47 μm), were increased by 14.5% than controls (*p* < 0.05; Fig. 4b). Cilia length of KCNH1^L352V^ did not show significant differences compared to wild-type (Fig. 4a and b).

On morphological evaluation of primary cilium structure, using ADP ribosylation factor like GTPase 13B (ARL13B) as cilium marker, we observed marked structural and numerical cilia defects in some mutant fibroblasts, with segmented axoneme and bulbous distal tips in KCNH1^R330Q^ fibroblasts and multiple cilia/cell in KCNH1^L352V^ fibroblasts (Fig. 4c). Similar structural defects were observed in hTERT RPE-1 cells overexpressing Flag-KCNH1 constructs carrying R330Q or L352V mutations (Supplementary Fig. 3; 4). No structural or numerical anomalies were detected in wild-type fibroblasts.

The multiple primary cilium defects of KCNH1^R330Q^ and KCNH1^L352V^ mutants suggest a possible perturbation of transport along the cilium. To evaluate this aspect, we analyzed the localization of Intraflagellar Transport 172 (IFT172), a member of the IFT-B complex, involved in anterograde transport along cilium [45]. Both KCNH1^R330Q^ and KCNH1^L352V^ mutant fibroblasts displayed a marked accumulation of IFT172 around the base of the cilium and at the tip, with apparent bulging (Fig. 4d and e) compared to a staining pattern around the base and along the entire shaft of the cilium in control cells. The observed bulbous morphology suggests an impaired retrograde transport, as observed in several cilia-related mutants [46].

### KCNH1^R330Q^ and KCNH1^L352V^ Mutations Induce Basal SHH Pathway Activation

**Fig. 5:**
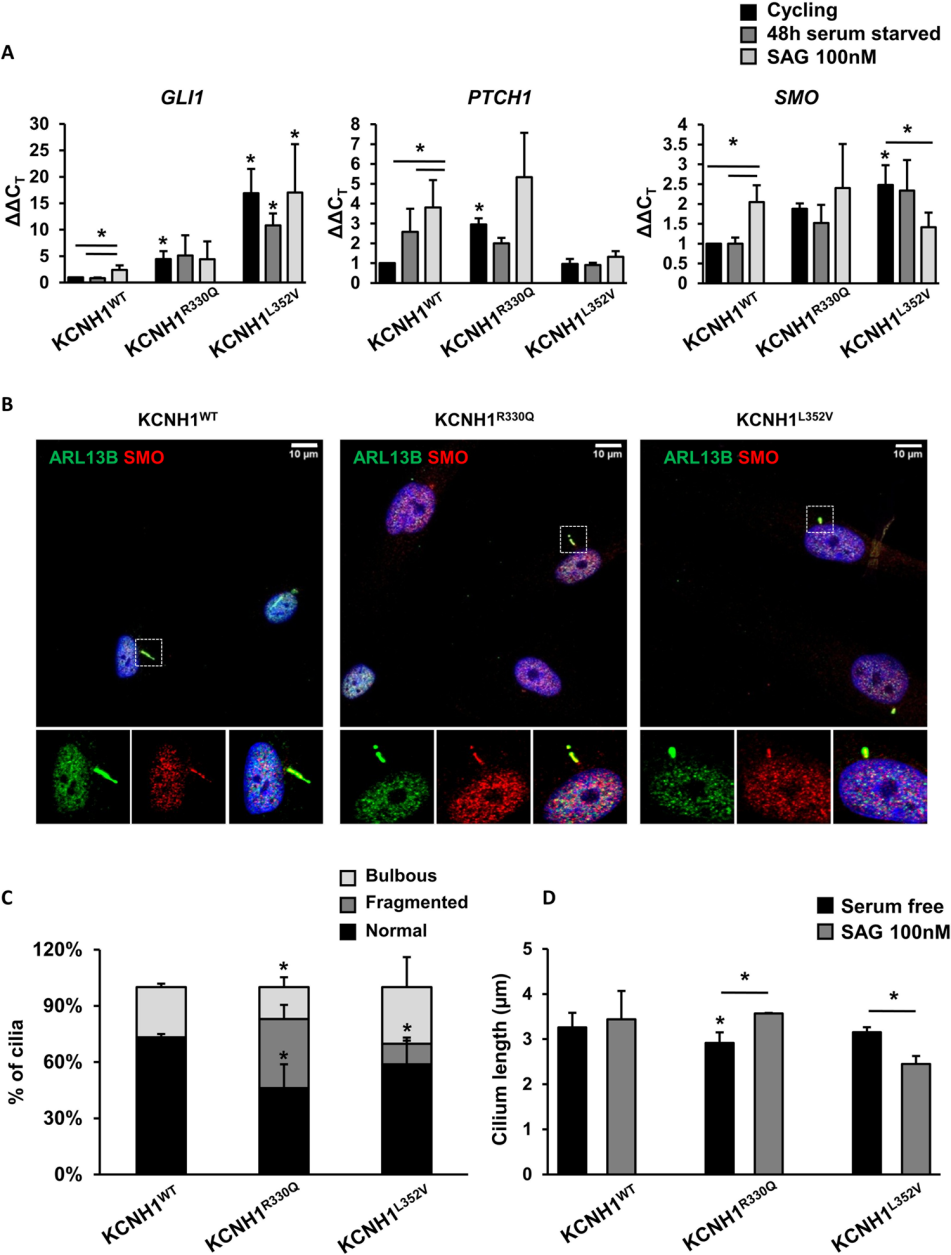
KCNH1 mutations induce basal SHH pathway activation. (**a**) Histograms show the expression levels of target genes of the SHH pathway (*GLI1*, *PTCH1*, and *SMO*) in fibroblasts from patients carrying KCNH1^R330Q^ and KCNH1^L352V^ mutations compared to control cells. Fibroblasts were serum-starved for 48 h and treated with SAG 100 nM. *GAPDH* was used as a reference gene for normalization. Data are represented as mean + sem. Differences between groups were analyzed by one-tailed Student’s *t* test. *p < 0.05 is reported vs the same condition of wild-type unless otherwise indicated with bars. (**b**) Representative images of IF analysis of ciliary localization of SMO (red) and ARL13B (green) in SAG treated wild-type and patients’ fibroblasts carrying KCNH1^R330Q^ and KCNH1^L352V^ mutations. Scale bars 10 µm. (**c**) Percentage of cells with cilia of different morphology (normal, fragmented, and bulbous). (**d**) Graph of cilia length measurements of KCNH1^WT^, KCNH1^R330Q^, and KCNH1^L352V^ fibroblasts in control and SHH pathway activation condition. Data are represented as mean + sem. Differences between two groups were analyzed by unpaired Student’s *t* test. *p < 0.05 is reported vs the same condition of wild-type unless otherwise indicated with bars

To evaluate the possible effect of KCNH1^R330Q^ and KCNH1^L352V^ mutations on the SHH signaling pathway transduced by the primary cilium [47], we analyzed gene expression of the SHH-responsive genes GLI Family Zinc Finger 1 (*GLI1*), *SMO*, and Patched 1 (*PTCH1*). As expected, the expression of *GLI1*, *SMO*, and *PTCH1* in wild-type cells significantly increased upon SAG treatment, a SMO agonist and a SHH pathway activator. Differently, in KCNH1 mutant fibroblasts, a marked and significant increase of those genes was detected already at basal conditions (*p* < 0.05; Fig. 5a).

SMO localization pattern in the cilium structure in the presence of SAG showed that SMO localizes along primary cilium in both wild-type and mutated fibroblasts upon SAG activation (Fig. 5b).

In SAG stimulated cells, a significant number of KCNH1^R330Q^ (37%; *p* < 0.05) and KCNH1^L352V^ (11%; *p* < 0.05) fibroblasts cilia compared to wild-type showed an altered morphology, characterized by a fragmented axoneme (*p* < 0.05; Fig. 5c). Moreover, when looking at cilia length, we observed a significant difference for both mutant fibroblasts compared to the cells without SAG treatment (*p* < 0.05; Fig. 5d). These results suggest that mutants’ cilia anomalies might be at least amplified by SHH pathway stimulation.

## Discussion

In this work, we studied the localization and function of the human KCNH1 (MIM *603305), a voltage-activated potassium channel, in human dermal fibroblasts and hTERT RPE-1 cells. We characterized the effect of pathogenic missense mutations on ciliogenesis and SHH pathway regulation, unraveling a multifaceted role of the protein.

Recently, KCNH1 has been localized to primary cilium during the G2/M transition of mammalian cells, and a role in cilium disassembly and cell proliferation has been preliminarily demonstrated [24]. In that work, the unexpected location of the full-length KCNH1, which is supposed to be a transmembrane protein, in the centrosomal component was explained as a residual ciliary membrane vesicle or, alternatively, attributed to the localization in the membranes of vesicles at the pre-ciliary compartment. Our immunofluorescence analyses support the localization of KCNH1 within the primary cilium and further refine the localization in the ciliary pocket structure and pre-ciliary vesicles, thus providing an explanation of the results mentioned above. Indeed, we demonstrated a clear colocalization with EHD proteins, four known components of pre-ciliary vesicles and ciliary pocket [48] since the early stages of ciliogenesis. EHD proteins coordinate crucial stages at the onset of cilium assembly [49]. Specifically, EHD1, the most ubiquitary member of the family, is well known to localize to pre-ciliary membranes, regulating their centrosomal trafficking, and is indispensable for ciliary vesicles’ formation and recruitment of transition zone proteins and IFT transport machinery at the M-centriole during early cilium assembly, facilitating fusion of the ciliary vesicles from distal appendage vesicles [49].

The ciliary pocket is a specialized membrane originated from an invagination of the ciliary membrane near the proximal part of the cilium and with crucial signaling functions. In particular, this region regulates ciliary membrane homeostasis and the assembly of multicomponent signaling complexes, representing a major site for vesicles’ exo- and endocytosis [6]. Hence, it could be hypothesized that KCNH1, besides the known subcellular localization, localizes in early cilium membrane structures where it could play a role in regulating early ciliary functions in primary cilium assembly/disassembly dynamics. This was supported by the occurrence of abnormal cilia morphology that we observed in human mutant fibroblasts, with a significant percentage of cells carrying cilia with dysmorphic features.

Mutations in *KCNH1*, whose oncogenic properties were reported in several studies and which is largely overexpressed in different cancers [50, 51], have been recently reported as causative of developmental diseases. To date, 45 patients with 24 *KCNH1* mutations have been reported with a phenotype characterized by a remarkable clinical heterogeneity, from mild to severe developmental delay and/or ID and epilepsy [27–39, 52].

Those phenotypes include Zimmermann-Laband syndrome 1 (ZLS1, MIM #135500), Temple-Baraitser syndrome (TMBTS, MIM #611816), syndromic intellectual disability and syndromic developmental delay, hypotonia and seizures. More recent studies broaden the phenotype spectrum to epilepsy and also to severe intellectual disability, mild extra-neurological phenotype, but lacking the distinctive features of TMBTS and ZLS1. Emerging evidence suggests a lack of correlation between the location of the affected residues and the clinical diagnosis [27, 33].

Most functional studies aimed to characterize potentially deleterious missense mutations, focused on channel electrophysiological properties, using patch-clamp electrophysiology experiments on Chinese hamster ovary (CHO) cells [27], Xenopus oocytes, and human HEK293T cells [30]. For nine missense mutations, a gain of function effect has been suggested based on a remarkable shift in the activation threshold to more negative potentials, producing dramatic increases in whole-cell K + conductance in the negative-potential range [27, 30].

In our study, we selected two pathogenic mutations localizing in different domains of the channel, L352V and R330Q (NP_002229.1). The L352 residue maps in the S5 pore helix [27], patch-clamp experiments showed strong negative shifts in voltage-dependent activation [27], and homology model analyses disclosed that the Leucine 352 residue forms a tight hydrophobic cluster with I467 and V356 in the open structure of the channel, which rearranges in the closed conformation suggesting that perturbations of these residues could affect the closed-open transition. The L352V mutation has been associated with ZLS1 in one patient with ID, seizures, coarse face, gingival enlargement, hypoplastic nails and terminal phalanges, scoliosis, and hypertrichosis [27].

The Arginine 330 residue localizes in the voltage-sensing domain (S4) of the channel, and the R330Q mutation has been reported in 7 cases [28, 33, 34, 37], with a phenotype including developmental delay, severe ID, absence/hypoplasia of great toe nail, neonatal hypotonia, gingival hyperplasia, and epilepsy starting in infancy. A further mutation was reported in the same residue (R330P) in one patient with syndromic developmental delay and infantile seizures [34]. No functional data are available on the R330P and R330Q variants that currently involve the most frequently mutated residue of KCNH1.

We observed that both considered pathogenic variants impaired ciliary structures, causing decreased length, fragmented axoneme, bulbous tips, and a multiciliated cell phenotype, with different degrees, as reported for several ciliopathies. Shorter cilia with abnormally bulged tips have been, in fact, associated with dynein-2 complex and centrosome proteins defects [40, 53], while cells with mutations in Meckel syndrome type 1 protein (*MKS1*) and Meckel syndrome type 3 (*MKS3*) genes causing Meckel syndrome show a multiciliated phenotype [54].

We might speculate that R330Q and L352V mutations cause different alterations in the primary cilium morphology, which may partially converge to the same pathways as IFT transport and SHH pathways, thus correlating with quite overlapping clinical phenotypes. Indeed, we demonstrated that both mutations cause a defective accumulation at the ciliary tip of IFT172, a component of the ciliary anterograde transport IFTB complex, and ectopic activation of the SHH signaling [24], a key pathway regulated by the ciliary pocket [6] and whose dynamics are closely reliant on IFT machinery [55, 56]. We suggest that observed altered SHH activation could be a consequence of the observed IFT172 mislocalization, probably causing abnormal ciliary trafficking. SHH pathway stimulation would induce additional morphological defects of cilia, which we observed in both mutants that showed excision of primary cilia tips. This feature resembles the recently discovered process named ciliary decapitation, used by primary cilia to release vesicles into the extracellular environment to promote quiescence exit through mitogenic SHH signaling [57], thus correlating with aberrantly SHH signaling activation.

KCNH1 confers accelerated cancer progression through molecular mechanisms such as regulation of cell cycle, proliferation, and ciliogenesis [51]. In this regard, conformational changes accompanying gating constitute a significant part of the KCNH1 oncogenic signal. Our results showed that KCNH1 mutations favoring the opening state induced ciliary SHH signaling and a significant increase of ciliated cells compared to wild-type protein without the induction of quiescent state. Increased ciliogenesis correlates with observed upregulation of *BCL2* expression, which has an anti-proliferative function and facilitates G0 arrest [43] so that we might speculate that a significant fraction of mutant fibroblasts is in a different phase of the cell cycle, possibly G0, compared to control fibroblasts.

On the contrary, by using Ki-67 as a proliferation marker, we observed that KCNH1 mutations increased the fraction of ciliated-proliferating cells, reducing the coupling between ciliogenesis and cell cycle and supporting previous results obtained in *KCNH1*-knockdown cells [24]. We also showed that, upon reintroduction of serum, cilium disassembly is impaired in fibroblasts with KCNH1^L352V^ mutation, as observed previously in *KCNH1*-knockout cells [24].

These findings suggest a role of voltage-gated potassium channels as regulators of ciliogenesis, possibly through the influencing of membrane function or vesicle transport adjacent to the transition zone or at the ciliary pocket, as firstly reported for Potassium Voltage-Gated Channel Subfamily Q Member 1 (KCNQ1), Potassium Inwardly Rectifying Channel Subfamily J Member 10 (KCNJ10), Potassium Voltage-Gated Channel Modifier Subfamily F Member 1 (KCNF1), and Chloride Voltage-Gated Channel 4 (CLCN4) [58], which localize to primary cilia, exert a role in ciliogenesis, and, when mutated (KCNQ1), cause defects in cilia structure, resulting in ciliopathy phenotypes in vitro [58].

KCNH1 has been proposed as a regulator of cilia assembly/disassembly dynamics through the modulation of Ca2 + concentration and phosphatidylinositol-4,5-bisphosphate (PIP2) accumulation at the base of the cilium, allowing lateral diffusion of ciliary components [59]. The pathogenic KCNH1 variants would confer a gain of function effect with increased channel activity that, in turn, would induce primary cilia disassembly and cause altered cilia structure [24]. Some clinical features of *KCNH1*-related phenotypes, as facial and digital malformations, which are included in the phenotype spectrum of some ciliopathies, have been considered as caused by perturbations of signaling pathways, as the SHH, which is involved in morphogenesis processes [24, 59]. Further functional studies are required to clearly link ciliary phenotypes to clinical phenotypes caused by specific gain-of-function *KCNH1* mutations impacting different domains.

The phenotypic spectrum of *KCNH1* pathogenic mutations includes a wide range of symptoms, from syndromic neurodevelopmental disorders to epilepsy. To date, epilepsy is the most recurrent disorder among the subjects carrying a *KCNH1* mutation and, interestingly, 3 cases of somatic variants were reported in subjects with epilepsy, one detected in the resected brain tissue of a patient with focal cortical dysplasia [39] and in two subjects, mothers of children with TMBTS, who have epilepsy but are otherwise healthy [30].

Epilepsy is reported in subsets of ciliopathy patients indicating the occurrence of neural circuit dysfunction as a consequence of primary cilia anomalies [60, 61]. Several observations indicate that primary cilia signaling are involved in the development and differentiation of cortical progenitors, neurons, and glia and that perturbation of this mechanism could underlie neurobehavioral or epilepsy phenotypes [14]. Therefore, it could be hypothesized that this phenotype could partly correlate to primary cilia signaling dysfunction in those cells.

The recent identification of gain-of-function mutations in two other K^+^ channels in individuals with syndromic developmental disorders (Potassium Two Pore Domain Channel Subfamily K Member 4 (*KCNK4*) in Facial Dysmorphism, Hypertrichosis, Epilepsy, Intellectual/Developmental Delay, and Gingival Overgrowth (FHEIG) syndrome and Potassium Calcium-Activated Channel Subfamily N Member 3 (*KCNN3*) in Zimmermann-Laband syndrome-3 (ZLS3) [62, 63]) led to suggest a new subgroup of rare potassium channelopathies, which comprises ZLS1, ZLS3, TMBTS, and FHEIG syndromes, caused by mutations in three genes *KCNH1*, *KCNN3*, and *KCNK4*, with *KCNH1* representing the most frequently mutated channel.

This prompted the interest in cellular dysfunctions related to potassium channels’ alterations causing disorders with overlapping clinical features, as developmental delay, ID, coarse facial features, gingival hypertrophy, nail and digital hypoplasia, and hypertrichosis [27].

As the biological mechanisms through which potassium channels mutations cause complex developmental phenotypes are still poorly characterized, the study of KCNH1 localization and its functions related to primary cilia and the alterations introduced by mutations in ciliogenesis, cell cycle coordination, cilium morphology, and cilia signaling pathway could help elucidate the molecular mechanisms underlying neurological phenotypes and neurodevelopmental disorders not considered as classical ciliopathies, but for which a significant role of primary cilia is emerging.

## Supplementary Information

Below is the link to the electronic supplementary material.

Supplementary file1 (DOCX 1259 kb)

Supplementary file2 (XLSX 11 kb)

Supplementary file3 (MP4 932 kb)

## Data Availability

Authors confirm that all relevant data are included in the article and its supplementary information files. Any other data that support the findings discussed here are available from the corresponding author upon reasonable request.
