# Supplementary material for: Potassium Channel KCNH1 Activating Variants Cause Altered Functional and Morphological Ciliogenesis
Source: Mol Neurobiol. 2022 May 31;59(8):4825–38. doi: 10.1007/s12035-022-02886-4 (PMC9363390; doi:10.1007/s12035-022-02886-4)

**Supplemental Material**

***Molecular Neurobiology***

**Potassium channel KCNH1 activating variants cause altered functional and morphological ciliogenesis**

Giulia Napoli ^1^, Noemi Panzironi ^2^, Alice Traversa ^2^, Caterina Catalanotto ^3^, Valentina Pace ^4^, Petrizzelli Francesco ^4,5^, Agnese Giovannetti ^2^, Sara Lazzari ^4^, Carlo Cogoni ^3^, Marco Tartaglia ^6^, Massimo Carella ^7^, Tommaso Mazza ^5^, Antonio Pizzuti ^4^, Chiara Parisi ^1^, Viviana Caputo ^4^.

1. CNR-National Research Council, Institute of Biochemistry and Cell Biology, Monterotondo Scalo, Rome, Italy.
2. Laboratory of Clinical Genomics, Fondazione IRCCS Casa Sollievo della Sofferenza, San Giovanni Rotondo (Foggia), Italy.
3. Department of Molecular Medicine, Sapienza University of Rome, Rome, Italy.
4. Department of Experimental Medicine, Sapienza University of Rome, Rome, Italy.
5. Unit of Bioinformatics, Fondazione IRCCS Casa Sollievo della Sofferenza, San Giovanni Rotondo (Foggia), Italy.
6. Genetics and Rare Diseases Research Division, Ospedale Pediatrico Bambino Gesù, IRCCS, Rome, Italy.
7. Research Unit of Medical Genetics, Fondazione IRCCS Casa Sollievo della Sofferenza, San Giovanni Rotondo (Foggia), Italy.

Corresponding authors:

Viviana Caputo: *viviana.caputo@uniroma1.it*

Parisi Chiara: *chiara.parisi@cnr.it*

**Supplementary methods**

**Constructs preparation, mutagenesis and cell transfection**

The human KCNH1 coding sequence corresponding to the short isoform (NM_002238.4, NP_002229.1, 962 amino acids) was amplified from human fetal cDNA using the following primers: KCNH1 Forward 5’-GTTTCCTGCTGTCGTAAGAAGC-3’; KCNH1 Reverse 5’-TGTTGGTCATGTGGACATATGTG-3’. The PCR products were gel-purified using the Wizard SV Gel and PCR Clean-Up System kit (Promega) and cloned in pSC-A-amp/kan vector using the Strataclone TA Cloning Kit (Agilent) according to the manufacturer’s protocol. Positive colonies were amplified, and plasmid DNA purified with Wizard Plus SV Minipreps DNA Purification System kit (Promega) was subjected to Sanger sequencing.

Then, the fragment corresponding to the KCNH1 isoform was cloned from pSC-A-amp/kan vector to the eukaryotic expression vector pCMV-TAG2A using the restriction enzymes sites BamHI and SalI. Missense mutations L352V and R330Q, were introduced by site-directed mutagenesis, using Pick Mutant Site-directed Mutagenesis kit (Canvax) in accordance with the manufacturer's protocol. All constructs were checked by direct sequencing.

hTERT RPE-1 cells were transfected with vectors expressing wild-type or mutants Flag-tagged KCNH1, respectively, using Lipofectamine 3000. After 48h of transfection cells were fixed with 4% paraformaldehyde (20 min at RT). After permeabilization with 0.1% TRITON X-100 (10 min at room temperature), non-transfected cells were stained with indicated primary antibodies.

**Supplementary Fig.1** KCNH1 partially localizes to Golgi reticulum and early endosomes in wild-type fibroblasts. Representative images of IF microscopy analysis of GOLGIN or RAB5 (green) and KCNH1 (red) distribution in wild-type fibroblasts. DAPI (blue) was used to visualize nuclei. Scale bars 10 µm.


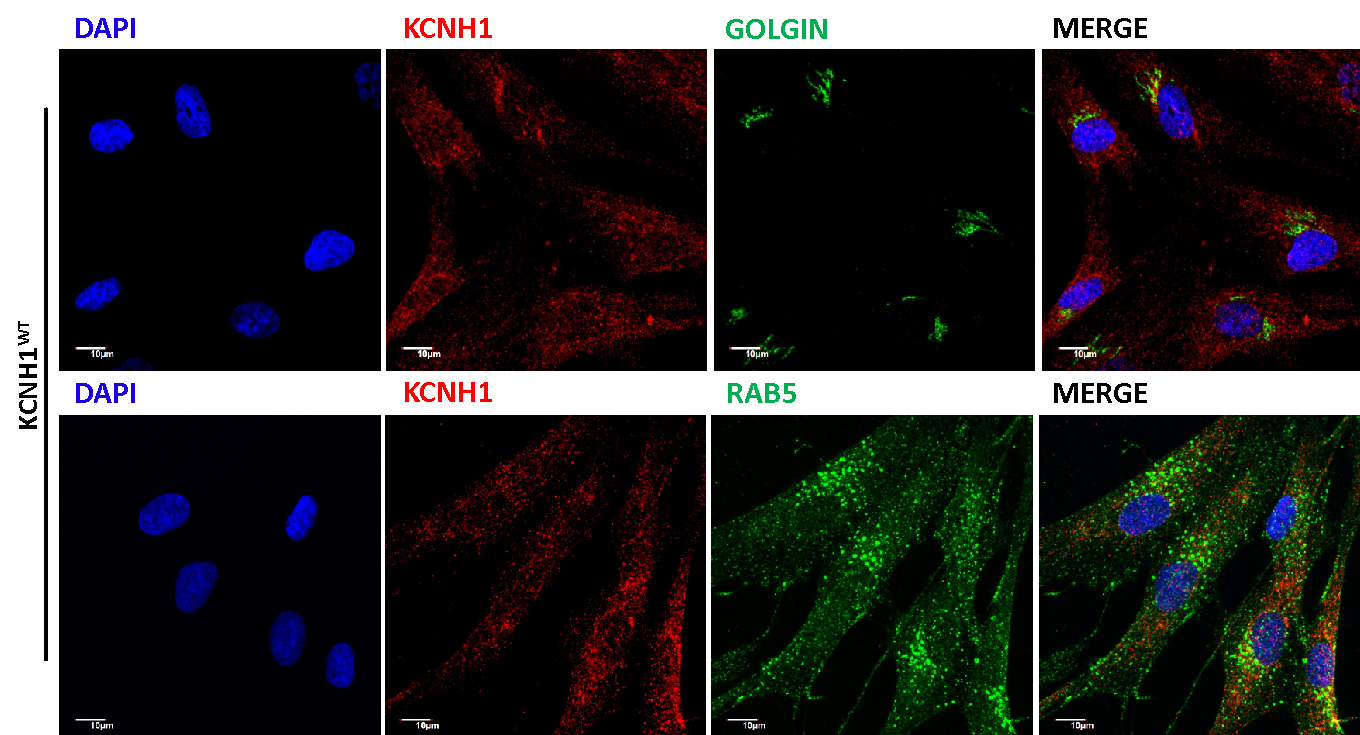


**Supplementary Fig.2** Exogenous Flag-tagged KCNH1 localizes to the primary cilia. hTERT RPE-1 cells were transfected with plasmid expressing KCNH1 Flag-tagged wild-type, R330Q and L352V mutant proteins. The staining was performed using Flag (green), acetylated tubulin (Ac. Tub) (red) and CEP164 (pink) antibodies. The panel shows localization of KCNH1 to the primary cilium base (white arrow). Scale bars 10 µm.


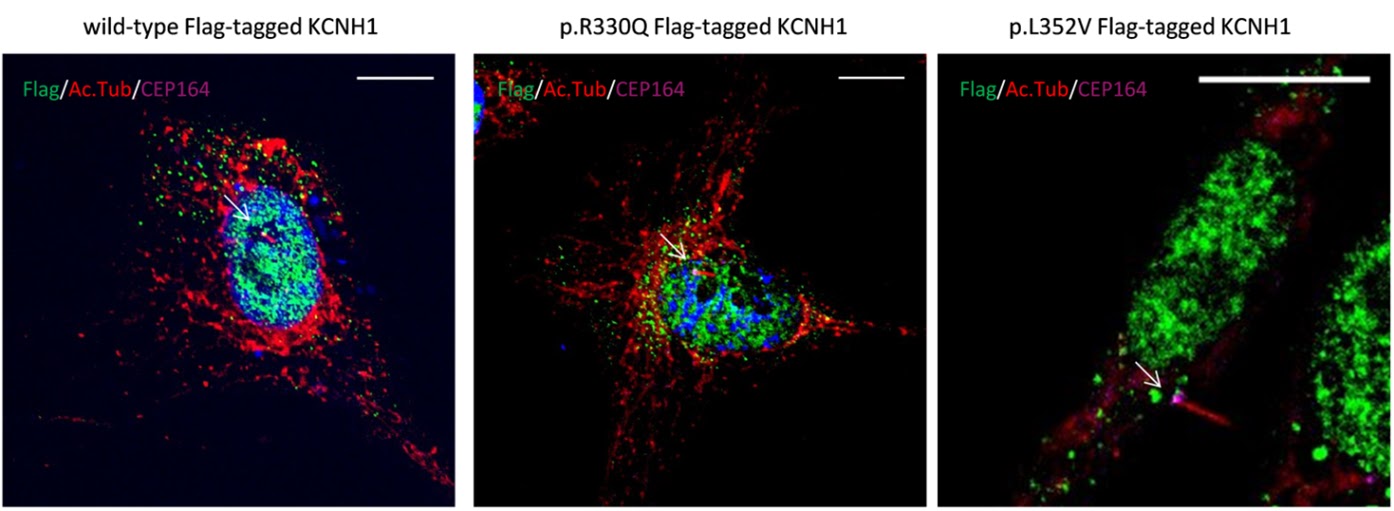


**Supplementary Fig.3** R330Q KCNH1 mutation affects cilia morphology in quiescent cells. (a) Immunofluorescence microscopy of ciliated wild-type and KCNH1^R330Q^ fibroblasts stained with antibody to ARL13b (red). DNA was visualized with DAPI (blue). KCNH1^R330Q^ fibroblasts show dysmorphic features (bulbous tip and discontinuous axoneme) compared to the control. Scale bars 10 µm. (b) Immunofluorescence microscopy of ciliated hTERT RPE-1 cells transfected with plasmid expressing KCNH1 Flag-tagged expressing wild-type and R330Q mutant proteins. The staining was performed using acetylated tubulin (Ac.Tub) (red) and CEP164 (pink) antibodies and DNA was visualized with DAPI (blue). R330Q mutation leads to formation of bulbous ciliary tips. Scale bars 10 µm.


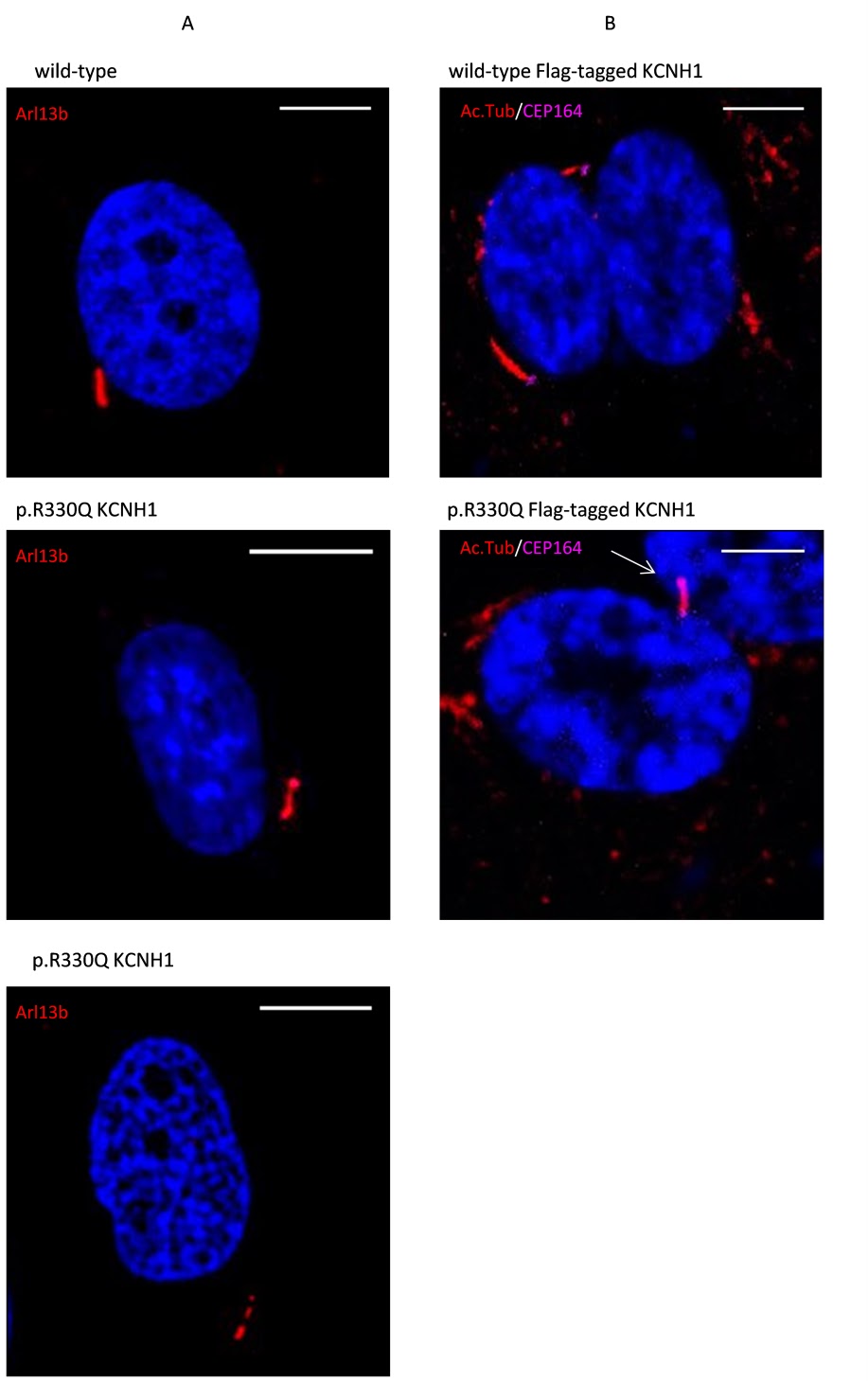


**Supplementary Fig.4** KCNH1 L352V variant leads to multiple cilia formation. (a) Immunofluorescence microscopy of ciliated wild-type and KCNH1^L352V^ patient’s fibroblasts stained with antibodies to acetylated tubulin (Ac Tub) (green) and CEP170 (red). DNA was visualized with DAPI (blue). Patient’s fibroblasts exhibit multi-ciliated cells. Scale bars 10 µm. (b) Immunofluorescence microscopy of ciliated hTERT RPE-1 cells transfected with plasmid expressing KCNH1 Flag-tagged expressing wild-type and L352V mutant protein. The staining was performed using ARL13b (red) antibody and DNA was visualized with DAPI (blue). hTERT RPE-1 cells transfected with Flag-tagged KCNH1^L352V^ shows multiciliation. Scale bars 10 µm.


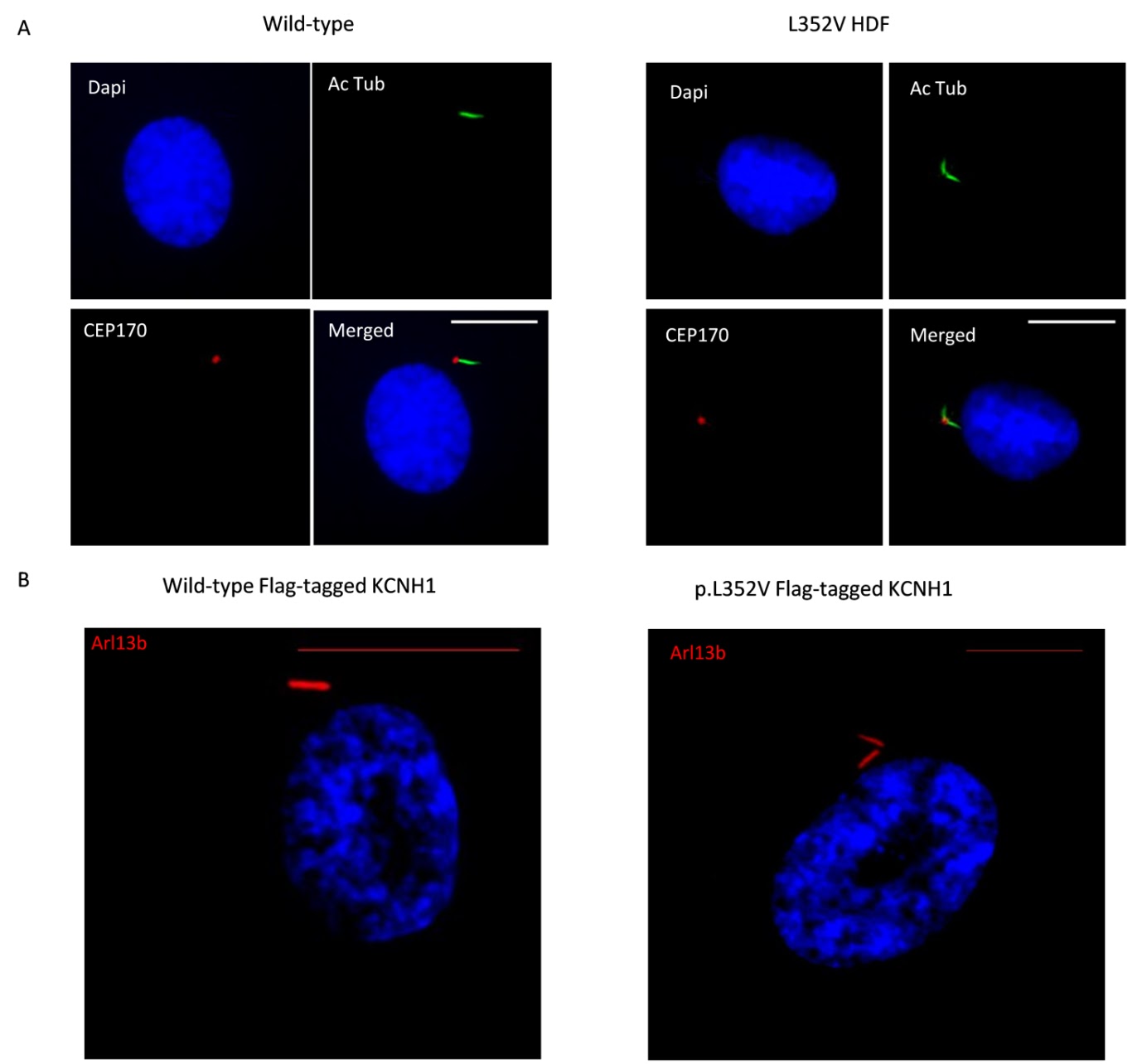

Supplement: Supplementary file 1 — Supplementary file1 (DOCX 1259 kb) [file 12035_2022_2886_MOESM1_ESM.docx]
